# Applying artificial vision models to human scene understanding

**DOI:** 10.3389/fncom.2015.00008

**Published:** 2015-02-04

**Authors:** Elissa M. Aminoff, Mariya Toneva, Abhinav Shrivastava, Xinlei Chen, Ishan Misra, Abhinav Gupta, Michael J. Tarr

**Affiliations:** ^1^Center for the Neural Basis of Cognition, Carnegie Mellon UniversityPittsburgh, PA, USA; ^2^Department of Psychology, Carnegie Mellon UniversityPittsburgh, PA, USA; ^3^Department of Machine Learning, Carnegie Mellon UniversityPittsburgh, PA, USA; ^4^Robotics Institute, Carnegie Mellon UniversityPittsburgh, PA, USA

**Keywords:** scene processing, parahippocampal place area, retrosplenial cortex, transverse occipital sulcus, computer vision

## Abstract

How do we understand the complex patterns of neural responses that underlie scene understanding? Studies of the network of brain regions held to be scene-selective—the parahippocampal/lingual region (PPA), the retrosplenial complex (RSC), and the occipital place area (TOS)—have typically focused on single visual dimensions (e.g., size), rather than the high-dimensional feature space in which scenes are likely to be neurally represented. Here we leverage well-specified artificial vision systems to explicate a more complex understanding of how scenes are encoded in this functional network. We correlated similarity matrices within three different scene-spaces arising from: (1) BOLD activity in scene-selective brain regions; (2) behavioral measured judgments of visually-perceived scene similarity; and (3) several different computer vision models. These correlations revealed: (1) models that relied on mid- and high-level scene attributes showed the highest correlations with the patterns of neural activity within the scene-selective network; (2) NEIL and SUN—the models that best accounted for the patterns obtained from PPA and TOS—were different from the GIST model that best accounted for the pattern obtained from RSC; (3) The best performing models outperformed behaviorally-measured judgments of scene similarity in accounting for neural data. One computer vision method—NEIL (“Never-Ending-Image-Learner”), which incorporates visual features learned as statistical regularities across web-scale numbers of scenes—showed significant correlations with neural activity in all three scene-selective regions and was one of the two models best able to account for variance in the PPA and TOS. We suggest that these results are a promising first step in explicating more fine-grained models of neural scene understanding, including developing a clearer picture of the division of labor among the components of the functional scene-selective brain network.

## Introduction

The past several decades have given us an unprecedented view of the inner workings of the human brain, allowing us to measure localized neural activity in awake, behaving humans. As cognitive neuroscientists, our challenge is to make sense of this rich source of data, connecting the activity we observe to mental mechanisms and behavior. For those of us who study high-level vision, making this connection is particularly difficult—vision scientists have not yet articulated any clear theories about what constitutes a “vocabulary” of intermediate visual features or what are the underlying building blocks of scene or object representation. Here we begin to address this issue by taking a different path to articulating a candidate set of features for visual representation: using a variety of extant computer vision models that make different commitments as to what counts as a visual feature as proxies for models of biological vision. We suggest that, to the extent that computer vision models and biological vision systems have similar end goals, the two domains will overlap in both their representations and processing assumptions.

To explore this issue, we had participants view 100 different scenes while we measured their brain activity, using functional Magnetic Resonance Imaging (“fMRI”), in regions that are known to be preferentially involved in scene processing. In particular, we hold that meaningful information can be extracted from the reliable patterns of activity that occur within scene selective regions: the parahippocampal/lingual region (the parahippocampal place area, “PPA”), the retrosplenial complex (“RSC”), and the occipital place area (also referred to as the transverse occipital sulcus, “TOS”). However, due to a lack of any fine-grained theories of scene understanding, it is unclear as to how one goes about interpreting the complex meaning inherent in these neural patterns. As alluded to above, we turn to models of computer vision to help us unravel how the human brain encodes and represents visual scenes, directly comparing the representations of scenes within these artificial vision systems to our obtained patterns of BOLD activity as measured by fMRI. The application of models derived from computer vision has one significant advantage: the models are well specified. As such, any particular model makes clear and explicit assumptions regarding representation and correspondence between a model and human neural responses or behavior allows us to infer that the two work similarly. Hence our emphasis on comparing a large number of models that all work somewhat differently from one another. In adopting modern computer vision models, we also note that these systems are built to understand the same complex visual world we deal with everyday (i.e., in contrast to earlier models that relied on “toy” worlds or highly-restricted visual domains). In particular, some of the models we include leverage large-scale/“web-scale” image datasets that may more accurately learn informative visual regularities embedded in the natural environment.

In that we have no strong *a priori* knowledge as to which of several very different models might be most effective with respect to accounting for neural data, our primary goal is to test whether we observe some correspondence between the patterns of neural activity elicited in high-order visual scene regions (i.e., PPA, RSC, and TOS) and the patterns of scene similarity as defined by these varying artificial vision models, and, specifically, which of these models does the best job at accounting for the neural data. We are also interested in the correspondence between artificial and biological vision systems, as well as the correspondence between the patterns of similarity obtained from neural responses and from behaviorally-measured explicit perceptual ratings.

We should note that our focus on accounting for neural responses in three specific brain regions of interest—the PPA, RSC, and TOS—is based on several decades of research describing the neural responses of these particular regions. Each has been shown to be selectively responsive to and optimized for processing scenes as compared to other visual stimuli, for example, single objects, faces, and meaningless visual patterns. It is also the case that all three of these regions are involved both in scene perception and spatial navigation; however, the PPA tends to be preferentially involved in scene recognition and the RSC tends to be preferentially involved in processing the larger spatial environment (Epstein and Higgins, 2007). These regions have also been sensitive to scene parts: both objects and spatial relations (Harel et al., 2013; Park et al., 2014); as well as more global properties of a scene such as the spatial boundary (Kravitz et al., 2011; Park et al., 2011; Watson et al., 2014). Finally, PPA, RSC and TOS have been shown to carry information regarding the statistical significance of objects occurring with specific scene categories (Stansbury et al., 2013) and the PPA has been shown to be sensitive to mid-level visual features, for example, recurring textures (Cant and Goodale, 2011; Cant and Xu, 2012). However, despite this array of empirically-demonstrated sensitivities to properties of the visual world, the specific computations that give rise to these functional responses are not well understood.

Here we use models originating from the field of computer vision to help reveal the computational processes that may be realized within these scene-selective brain regions. Given that scenes are complex visual stimuli that carry useful information within low-level visual features (e.g., oriented lines, edges, junctions, etc.), mid-level features (e.g., groupings and divisions of features that are superordinate to the low-level features), and high-level features (e.g., semantic meaning, categorization) we apply several different computer vision methods to capture these multiple levels. In particular, we attempt to account for variation in our neuroimaging data collected while participants are viewing a wide variety of different scenes using both high-level semantic feature-based models (e.g., SUN semantic attributes; Patterson and Hays, 2012) and low-level visual feature-based models (e.g., SIFT, HOG; Lowe, 2004; Dalal and Triggs, 2005). We predict that low-level features will be encoded in brain areas that selectively process scenes, but are also encoded in non-scene-selective regions such as early visual areas. In contrast, as discussed below, mid- and high-level features that capture the inherent meaning of a scene are predicted to be specifically encoded in scene-selective brain regions exclusively.

In studying scene or object understanding, the field faces a significant challenge: between visual input and semantics there is a significant gap in knowledge with respect to any detailed account of the mid- and high-level visual features that form the representation of visual information. That is, almost all theories of mid- and high-level visual representation rest on human intuition, providing little formal method for articulating the features underlying visual semantics or its precursors: mid-level visual features that are compositional in nature (Barenholtz and Tarr, 2007). For example, for us, distinguishing between a manmade and a natural scene is trivial and we typically account for our judgments by referring to semantic features within a scene (e.g., trees, buildings). However, there are also mid-level features (e.g., rectangular shapes) that are highly correlated with a scene's high-level semantics that may provide some insight into how the visual system can so readily understand the difference between manmade and natural. As one example, recent work suggests that the PPA responds preferentially to both simple rectilinear features and objects comprised of a predominantly rectilinear features (Nasr et al., 2014). This and other results hint that focusing on high-level semantics exclusively may miss critical elements of how scenes are selectively processed in the human brain. Relying on human intuition also suffers from the Titchenerian problem that introspection alone does not have access to the unconscious processing that makes up the bulk of our cognition. Thus, theories based largely on intuition almost surely miss identifying the bulk of visual features (or parts) that are critical in the neural representation of scenes. To address the need for mid-level, non-intuition-based visual features, one of the primary (and most interesting) computer vision models we apply is NEIL, the “Never Ending Image Learner” (www.neil-kb.com; Chen et al., 2013). NEIL is a large-scale (“web-scale”) image-analysis system that, using only “weak supervision,” automatically extracts underlying statistical regularities (e.g., both mid-level and high-level visual attributes) from natural scene images and constructs intuitively-correct scene categories. In doing so, NEIL both limits the need for the application of human intuition and allows for the simultaneous exploration of features at multiple levels of scene representation (i.e., low- to high-level). In applying NEIL, we asked whether the attributes that NEIL learns to characterize scenes give rise to a scene similarity space that correlates with a neurally-derived scene similarity space. Good correspondence between the two domains representing the same scenes would suggest that cortical vision is sensitive to some of the same statistical regularities—at a variety of levels—NEIL extracts to build a category structure for scenes.

In the past few years, a small number of studies have applied models drawn from computer vision to the question of neural representation in visual cortex. For the most part, this approach has focused on object recognition and examined a wide region of visual cortex, including low-level regions, V1–V3, mid-level regions, V4, and high-level regions, IT (Baldassi et al., 2013; Leeds et al., 2013; Khaligh-Razavi and Kriegeskorte, 2014; Yamins et al., 2014). However, to our knowledge, only one study has combined computer vision methods with neural scene understanding. In particular, Watson et al. (2014) examined how well low-level scene features derived from GIST, a descriptor that analyzes orientation energy at different spatial frequencies and spatial positions (Oliva and Torralba, 2001), might account for the fMRI-derived neural patterns associated with scene processing in the human ventral stream. They found that scene-specific regions (PPA, RSC, TOS) elicited patterns of activity that were better accounted for by low-level (GIST) properties as compared to semantic categories for scenes. However, Watson et al.'s study is limited by its “jump” from very low-level features (GIST) to very high-level semantic categories and their use of only four scene categories. Here we build on this result by looking at different metrics at different levels of representation and expanding the space of stimuli to 100 different scenes across 50 different scene categories, asking how well this range of computationally-motivated metrics can account for the complex neurally-derived scene space we measure in PPA, RSC, and TOS.

At the same time we explore representational metrics derived from computer vision, we also consider human behavior directly, examining the scene space derived from how humans judge two visually-presented scenes as similar. *A priori*, if two scenes are judged as similar, we might expect that the two scenes would elicit similar neural response patterns in scene selective brain regions. Of course, as noted earlier, explicit intuitions about cognitive processing are unreliable indicators of the complex underlying mechanisms supporting such processing. As such, it is unclear as to whether conscious behavior is a good predictor of neural representation. Thus, models of representation arising from computer vision may actually reveal more subtle information about neural encoding that cannot be inferred using behavioral methods. This empirical question—how well does behavior explain the neural activity elicited by scene understanding—is included in our study as a benchmark against which we can measure the performance of the computer vision models we apply to our data.

More generally, it is worth considering what we might be able to infer from our present methods. Particularly given our background emphasis on explicating better-specified accounts of mid- and high-level features, we might hope that a fine-grained analysis of our results would reveal the *specific* nature of representational features (e.g., a catalog of some sort). Unfortunately, such a detailed account is beyond what is realistically possible in our present study given: (1) the power limitations arising from the low number of observations we can collect from individual participants in an fMRI session; (2) the low SNR of BOLD responses; and (3) the middling spatial and poor temporal resolution of fMRI. To be clear, we view our present study as a first step in working toward such detailed accounts, but, realistically, such an account is not obtainable without many refinements in methods and theories. That being said, we hold that our present study does allow important inferences about the neural representation of scenes. More specifically, as discussed earlier, each of the computer vision models employed here makes assumptions regarding how it encodes visual scene information. Although the similarity metrics we use do not allow us to break down these assumptions to the level of specific features, they do help us choose between different models. Such model selection is common in some areas of science, but less so in the cognitive neurosciences where there are often few options from which to select (which is our point about the current state of knowledge regarding mid- and high-level visual representation). Our approach is to adopt a range of models from computer vision to enable a more comprehensive search space that encompasses a wider range of representational assumptions, including assumptions that might not be inferred through intuition. In the end, we learn something about which representational assumptions appear most promising for further investigation, thereby laying the groundwork for studies in which we specifically manipulate features derived from the most effective models.

A separate concern relates to a potential confound between receptive field (RF) size and feature complexity. At issue is the fact that more complex features tend to encompass more of the visual field and, therefore, are more likely to produce responses in the extrastriate scene-selective regions that are known to have larger RF sizes. However, we are less than certain as to how one would tractably partial out RF size from feature complexity. For example, if more complex features are more complex precisely because they are more global and reflect the relations between constituent parts, then—by definition—they are also captured in larger RFs. This is similar to the confound in the face recognition literature between RF size and “holistic” or “configural” processing (see for example, Nestor et al., 2008). Researchers argue that a particular effect is holistic, when, in fact, it is also the case that it is captured by larger RFs. Indeed, it may be that much of what we think of in the ventral pathway with respect to complexity is reasonably equivalent to RF size. We view trying to tease these two dimensions apart as an important question, but one that is beyond our present study.

More concretely, our study empirically examines human visual scene processing by way of scene similarity across three different domains: neuroimaging data, behavior, and computer vision models. In particular, we used fMRI with a slow event-related design to isolate the patterns of neural activity elicited by 100 different visual scenes. Using a slow event-related design we were able to analyze the data on a trial-by-trial/scene-by-scene basis, therefore allowing us to associate a specific pattern of BOLD activity with each individual scene. We then constructed a correlation matrix representing “scene-space” based on this neural data, performing all pairwise correlations between measured neural patterns within the brain regions of interest. This neurally-defined scene-space was then correlated with scene-spaces arising from a range of computer vision models [see Section Computer Vision (CV) Metrics]—each one providing a matrix of pairwise scene similarities of the same dimensionality as our neural data. At the same time, to better understand how the neural representation of scenes relates to behavioral judgments of scene similarity, we also ran a study using Amazon Mechanical Turk in which participants rated the similarity, on a seven-point scale, between two visually-presented scenes (4950 pairwise similarity comparisons).

## Materials and methods

### Stimuli

Scene stimuli were 100 color photographs from the NEIL database (www.neil-kb.com) (Chen et al., 2013) depicting scenes from 50 different scene categories as defined by NEIL—two exemplars from each category were used. Categories ranged from indoor to outdoor and manmade to natural in order to achieve good coverage of scene space. See Supplemental Material for a list of categories and Figure S1 for images of stimuli used. Scene images were square 600 × 600 pixels, and were presented at a 7° × 7° visual angle.

### fMRI experiment

#### Localizer stimuli

Stimuli used in the independent scene “localizer” consisted of color photographs of scenes, objects, and phase-scrambled pictures of the scenes. The objects used were not strongly associated with any context, and therefore were considered weak contextual objects (e.g., a folding chair) (Bar and Aminoff, 2003). Pictures were presented at 5° × 5° visual angle. There were 50 unique stimuli in each of the three stimulus conditions.

#### Participants

Data from nine participants in the fMRI portion of the study were analyzed (age: *M* = 23, 20–29; two left handed; five female). One additional participant (i.e., *N* = 10) was excluded from the data analysis due to falling asleep and missing a significant number of trial responses. Data from one other participant only had half the dataset included in the analysis due to severe movement issues in one of the two sessions. All participants had normal, or corrected-to-normal vision, and were not taking any psychoactive medication. Written informed consent was obtained from all participants prior to testing in accordance with the procedures approved by the Institutional Review Board of Carnegie Mellon University. Participants were financially compensated for their time.

#### Procedure

Each individual participated in two fMRI sessions in order to acquire sufficient data to examine the responses associated with individual scenes. Both sessions used the same procedure. The average time between the two sessions was 3.6 days, ranging from 1 to 7 days. Each fMRI session included six scene processing runs, a high resolution mprage anatomical scan run after the third scene processing run, and at the end of the session, one or two runs of a functional scene localizer.

During fMRI scanning, images were presented to the participants via 24 inch MR compatible LCD display (BOLDScreen, Cambridge Research Systems LTD., UK) presented at the head of the bore and reflected through a head coil mirror to the participant. Each functional scan began and ended with 12 s of a white fixation cross (“+”) presented against a black background. For the scene processing runs, there were 50 picture trials—one exemplar from each of the 50 categories. The paradigm was a slow event-related design and order of the stimuli were random within the run. Two runs were required to get through the full set of 100 scenes, with no scene category repeating within the run. There were three presentations of each stimuli in each session (i.e., six functional runs) and across the two sessions, there were data for a total of six trials per a unique stimulus. Stimuli were presented for 1 s, followed by 7 s of fixation. On a random eight of the 50 trials of a run, the image rotated a half a degree to the right and then back to center, which took a total of 250 ms. Participants were asked to press a button when a pictured “jiggled.” Participants performed on average 96% correct.

After all six of the scene processing runs, a functional scene localizer was administered in order to independently define scene selective areas of the cortex (PPA, RSC, and TOS). The localizer was a block design such that 12 stimuli of the same condition (either scenes, objects, or phase scrambled scenes) were presented in row. Each stimulus was presented for 800 ms with a 200 ms ISI. Between stimuli blocks, there were 8 s of a fixation cross presentation. There were six blocks per condition, and 18 blocks across conditions per run. The participant's task was to press a button if the picture immediately repeated (1-back task), of which there were two per block. Thus, in each block there were 10 unique stimuli presented, with two stimuli repeated once. Based on time of the scan session and energy of the participant, either one or two localizer runs were administered.

Before the participant went into the MRI scanner, they were told to remember the images as best as possible for a memory test. Once the participant concluded the fMRI portion of the session they performed a memory test outside the scanner. In the memory test, there were two trials for each of the 50 scene categories, with one trial presenting an image from the MRI session and the other trial presenting a new exemplar. For each trial, the participant had a maximum of 3 s to respond, with the picture on the screen for the entire time. The picture was removed from the screen as soon as the participant responded and the next trial began. Participants were 81% correct on average. The memory test was used to motivate the participants to pay attention, and was not used in any of the analyses.

#### fMRI data acquisition

Functional MRI data was collected on a 3T Siemens Verio MR scanner at the Scientific Imaging and Brain Research Center at Carnegie Mellon University using a 32-channel head coil. Functional images were acquired using a T2^*^-weighted echoplanar imaging pulse sequence (31 slices aligned to the AC/PC, in-plane resolution 2 × 2 mm, 3 mm slice thickness, no gap, *TR* = 2000 ms, *TE* = 29 ms, flip angle = 79°, GRAPPA = 2, matrix size 96 × 96, field of view 192 mm, reference lines = 48, descending acquisition). Number of acquisitions per run was 209 for the main experiment, and 158 for the scene localizer. High-resolution anatomical scans were acquired for each participant using a T1-weighted MPRAGE sequence (1 × 1 × 1 mm, 176 sagittal slices, *TR* = 2.3 s, *TE* = 1.97 ms, flip angle = 9°, GRAPPA = 2, field of view = 256).

#### fMRI data analysis

All fMRI data were analyzed using SPM8 (http://www.fil.ion.ucl.ac.uk/spm/) and in-house Matlab scripts. Data across the two sessions were realigned to correct for minor head motion by registering all images to the mean image.

***Functional scene localizer***. After motion correction, the data of the scene functional localizer was smoothed using an isotropic Gaussian kernel (FWHM = 4 mm). The data was then analyzed as a block design using a general linear model and a canonical hemodynamic response function. A high pass filter using 128 s was implemented. The general linear model incorporated a robust weighted least squares (rWLS) algorithm (Diedrichsen and Shadmehr, 2005). The model simultaneously estimated the noise covariates and temporal auto-correlation for later use as covariates within the design matrix. The six motion parameter estimates that output from realignment were used as additional nuisance regressors. Data were collapsed across all localizer runs, with each run used as an additional regressor. The design matrix modeled three conditions: scenes, weak contextual objects, and phase scrambled scenes. The main contrast of interest was examining the differential activity that was greater for scenes as compared with objects and phase-scrambled scenes.

***Event-related scene data***. After motion correction, the data from the scene task runs were analyzed using a general linear model. Motion corrected data from a specific region of interest was extracted and nuisance regressors from the realignment were applied. The data was subjected to a 128 s high pass filter and was subjected to correction from rWLS, as well as a regressor represented each of the different runs. The data for the entire event window (8 s) was extracted for each scene stimulus, for each voxel within the region of interest, and averaged across the number of repetitions. Data in the 6–8 s time frame was used for all further analysis. This was the average peak activity in the time course across all trials for all participants. All six presentations of the stimulus were averaged together, including those that “jiggled” for the 250 ms. A similarity matrix of all the scenes (100 × 100) was then derived by cross-correlating the data for each scene across the voxels in the brain regions of interest within each individual. *R*-values from each of the cells in the similarity matrix were then averaged across participants for a group average.

#### Region of interest (ROI) analysis

All regions of interest analyzed were defined at the individual level using the MarsBaR toolbox (http://marsbar.sourceforge.net/index.html). Scene-selective regions (PPA, RSC, and TOS) were defined using the localizer data in the contrast of scenes greater than objects and phase-scrambled scenes. Typically, a threshold of FWE *p* < 0.001 was used to define the set of voxels. Size of ROIs were *a priori* chosen to have a goal of 100–200 voxels, or as close to that as possible. Two control non-scene selective ROIs were also chosen. One was a region in very early visual cortex along the left hemisphere calcarine sulcus defined in the localizer data as phase-scrambled greater than objects. The right hemisphere dorsolateral prefrontal cortex (DLPFC) was also chosen as a control region, which was defined using the localizer data in an all task (collapsed across all three conditions) greater than baseline comparison. Typically the threshold for the DLPFC ROI was lower than the other ROIs—FWE *p* < 0.01, or *p* < 0.00001 uncorrected, if not enough voxels survived the correction. Control ROIs were defined in all participants.

### Amazon mechanical turk (MTurk)

Behavioral judgments of similarity for each pairwise comparison of scenes were acquired through the use of study administered on MTurk.

#### Participants

Participants were voluntarily recruited through the human intelligence task (HIT) directory on MTurk. Enough individuals were recruited to satisfy reaching 20 observations for each of the 4950 pairwise scene comparisons. This resulted in 567 individuals participating in at least one HIT (10 scene pairs). An individual participated in an average of 17.2 HITs, and the range was from 1 to 174. All participants reported they were over the age of 18, with normal or corrected to normal vision, and located within the United States. Participants were financially compensated for each HIT completed. Participants read an online consent form prior to testing in accordance with the procedures approved by the Institutional Review Board of Carnegie Mellon University.

#### Procedure and data analysis

Each HIT contained 11 comparisons. Pairs of scenes were presented side-by-side, and the participant was asked to rate the similarity of the two scenes on 1–7 scale (1 = completely different; 7 = very similar), there was also an option of 8 for identical. The scale was presented below the pair of images with both the number and the description by each response button. In each HIT there was one pair that was identical for use as a catch trial. Participants were encouraged to use the entire scale. A participant's data were removed from the analysis if he/she did not respond correctly on the catch trials. If the participant missed a number of catch trials (over the course of several HITs) and exclusively used only 1 and 8 on the scale, that participant's entire data was removed from the analysis due to ambiguity as to whether she/he was actually completing the task, or just pressing 1 and 8. All valid data was then log transformed due to a preponderance of different judgments relative to any other response; skewness of 2.67 (*SE* = 0.04) and kurtoisis of 8.76 (*SE* = 0.07). The data were then used to construct a similarity matrix of the scenes (100 × 100) with the value of each cell determined by the average response for the pairwise comparison across the ~20 observations. Some comparisons had missing responses due to removal of ambiguous data.

### Computer vision (CV) metrics

Each of the 100 scenes was analyzed by several different computer vision methods. The vector of features for each scene within each model was cross-correlated across all pairwise scene correlations to generate the similarity matrix defining the scene-space for that technique. We chose a wide variety of computer vision models that implement features that can roughly be divided in two categories: mid- and high-level attribute-based (NEIL, SUN semantic attributes, GEOM) and low-level (GIST, SIFT, HOG, SSIM, color). The former, attribute-based features, capture semantic aspects in the image, for example, highways, fountains, canyons, sky, porous etc. Low-level features such as GIST, SIFT, and HOG capture distributions of gradients and edges in the image. Gradients are defined as changes/derivatives of pixel values in the X and the Y direction in the image and edges are obtained after post-processing of these gradients. Note that for the purposes of this paper, we will use the terms gradients and edges interchangeably. Finally, models such as SSIM encode geometric layout of low-level features and shape information in the image. Local self-similarities in edge and gradient distributions complement low-level features such as those in SIFT. Critically, all of these models have a proven track record for effective scene classification (Oliva and Torralba, 2006; Vedaldi et al., 2010; Xiao et al., 2010). We now describe each of these models in more detail.

#### NEIL

The Never-Ending Image Learner (Chen et al., 2013) is a system that continuously crawls the images on the internet to automatically learn visual attributes, objects, scenes and common sense knowledge (i.e., the relationships between them). NEIL's strength comes from the large-scale data it analyzes in which it learns this knowledge; and by using commonsense relationships in this knowledge base to constrain its classifiers. NEIL's list of visual attributes were generated using the following mechanism (Chen et al., 2013): first, an exhaustive list of attributes used in the computer vision community were compiled, which included semantic scene attributes (SUN) (Patterson and Hays, 2012; Shrivastava et al., 2012), object attributes (Farhadi et al., 2009; Lampert et al., 2013) and generic attributes used for multimedia retrieval (Naphade et al., 2006; Yu et al., 2012). This exhaustive list was then pruned to only include attributes that represented adjectival properties of scenes and objects (e.g., red, circle shape, vertical lines, grassy texture). At the time of our study, the scene classifiers learned by NEIL were based on a scene space defined by 84 of these visual attributes, encompassing low-, mid- and high-level visual information of the scenes. For each scene there is a vector of scores, one for each attribute, of how confidently that attribute can be identified in that scene image. For each attribute classifier, we computed the variance of its scores across all scene categories used within the experiment, and used exponentiated variance for re-weighing the scores of each attribute individually. This normalization increases the weights on attributes that are more effective for distinguishing between scene categories and down weights the attributes that are less effective. The similarity matrix for NEIL was constructed as a cross-correlation of these scores.

#### Semantic scene attributes (SUN)

We use the set of 102 high-level SUN attributes as proposed in Patterson and Hays (2012), which were originally defined through crowd-sourcing techniques specifically intended to characterize scenes. These attributes were classified under five different categories: materials (e.g., vegetation), surface properties (e.g., sunny), functions or affordances (e.g., biking), spatial envelope (e.g., man-made), and object presence (e.g., tables). For each attribute, we have a corresponding image classifier as trained in Patterson and Hays (2012). The scores of these 102 classifiers were then used as features. These scores represent the confidence of each classifier in predicting the presence of the attribute in the image. The similarity matrix for SUN was constructed as a cross-correlation of these semantic attribute scores.

#### GEOM

Geometric class probabilities (Hoiem et al., 2007) for image regions—ground (gnd), vertical (vrt), porous (por), sky, and all were used. The probability maps for each class are further reduced to 8 × 8 matrix, where each entry represents the probability of the geometric class in a region of the image (Xiao et al., 2010). The similarity matrix for GEOM for each subset definition (e.g., vrt) was constructed as a cross-correlation of the probability scores for each region of the picture.

#### GIST

GIST (Oliva and Torralba, 2006) captures spatial properties of scenes (e.g., naturalness, openness, symmetry etc.) using low-level filters. The magnitude of these low-level filters encodes information about horizontal and vertical lines in an image, thus encoding the global spatial structure. As a byproduct, it also encodes semantic concepts like horizon, tall buildings, coastal landscapes etc., which are highly correlated with distribution of horizontal/vertical edges in an image. The GIST descriptor is computed using 24 Gabor-like filters tuned to 8 orientations at 4 different scales. The squared output of each filter is then averaged on a 4 × 4 grid (Xiao et al., 2010). The similarity matrix for GIST was constructed as a cross-correlation of these averaged filter outputs (512 dimensions).

#### HOG 2 × 2 (L0–L2)

Histogram of oriented gradients (HOG) (Dalal and Triggs, 2005) divides an image into a grid of 8 × 8 pixel cells and computes histogram statistics of edges/gradients in each cell. These statistics capture the rigid shape of an image and are normalized in different ways to include contrast sensitive, contrast insensitive and texture distributions of edges. For HOG 2 × 2 (Felzenszwalb et al., 2010; Xiao et al., 2010), the HOG descriptor is enhanced by stacking spatially overlapping HOG features, followed by quantization and spatial histograms. The spatial histograms are computed at three levels on grids of 1 × 1 (L0), 2 × 2 (L1) and 4 × 4 (L2) (see Xiao et al., 2010, for details). The similarity matrix for HOG 2 × 2 (L0–L2) was constructed as a cross correlation of these histogram features at different image regions and spatial resolutions.

#### SSIM (L0–L2)

Self-similarity descriptors (Shechtman and Irani, 2007) capture the internal geometric layout of edges (i.e., shape information) using recurring patterns in edge distributions. The descriptors are obtained by computing the correlation map of a 5 × 5 patch in a window with 40 pixels radius, followed by angular quantization. These SSIM descriptors are further quantized into 300 visual words using k-means (see Xiao et al., 2010, for details). The similarity matrix for SSIM was constructed as a cross correlation of these histograms of visual words at different spatial resolutions.

Finally, we included a variety of local image features based on image gradient/texture and color. Following the standard Bag-of-Words approach (vector quantization of features using k-means), we generated a fixed-length representation for each image. We used various dictionary sizes (*k* = 50, 250, 400, 1000) for each feature. For implementation, (van de Sande et al., 2011) was used for feature extraction and (Vedaldi and Fulkerson, 2010) for k-means quantization of features. As suggested by Vedaldi and Fulkerson (2010), we also L2 normalized each of the histograms. The similarity matrix for each of the local image features below was constructed as a cross correlation of these histograms of visual words for each local feature. The local features used were as follows:

- Hue histogram (50, 250, 400, 1000): A histogram based on the hue channel 1 of the image in the HSV color space representation. Roughly speaking, hue captures the redness/greenness/blueness etc. of the color.
- SIFT (50, 250, 400, 1000): Scale invariant feature transform (SIFT) (Lowe, 2004) characterizes each image based on local edge features. For each point in the image, it captures the gradient distribution around it, generally by computing histograms of edge feature in local neighborhood/patch and normalizing these histograms to make the descriptor rotationally invariant (even if the patch of pixels is rotated, the computed SIFT feature is the same). Standard SIFT works on grayscale images, and we use dense-SIFT (see Xiao et al., 2010; van de Sande et al., 2011).
- Hue-SIFT (50, 250, 400, 1000): SIFT computed only on the hue channel of the HSV representation of the input image.
- RGB-SIFT (50, 250, 400, 1000): SIFT computed on each color channel (R, G, and B) independently, and then concatenated.

### Correlations across measures

The similarity matrix arising from each method was converted into a vector using data from one side of the diagonal. This data were then fisher corrected for all analyses. First, a cross correlation analysis was performed to acquire the Pearson's *r* correspondence between each method. The *p*-values in this cross correlation are assumed to survive a Bonferroni correction correcting for 4950 pairwise correlations of scenes (*p* < 0.00001). For the regression analysis, *p*-values were corrected against 39 correlations (All ROIs, behavior, CV measures). To test the significance between model fits, a bootstrapping method was implemented. Testing across 1000 iterations of samples with replacement, a 95% confidence interval between model fits (*r*^2^) was defined. The confidence interval reflected a *p* < 0.05 correcting for multiple comparisons. If the difference between the model correlations exceeded the confidence interval, the models were considered significantly different from each other (Wasserman, 2004).

## Results

We examined scene encoding in the human visual cortex by defining ROIs in the brain that preferred scene stimuli to weak-contextual objects and phase-scrambled scenes. This gives rise to three ROIs: the PPA, RSC, and TOS where the BOLD signal was found to be significantly greater when viewing scenes as compared to objects or phase-scrambled scenes. Additional two brain regions were defined, an early visual region and a region in the dorsolateral prefrontal cortex (DLPFC, see Materials and Methods). These regions were chosen as control regions to compare the scene ROIs (PPA, RSC, and TOS) to regions of the brain involved in visual processing or in a cognitive task involving visual stimuli, but that are not believed to be specific to scene processing. Data for each of the 100 scenes were then extracted on a voxel by voxel basis for each ROI. To examine the encoding of scenes each pairwise correlation of the scenes was computed to determine how similar the patterns of activity across the voxels of an ROI were from scene to scene. The resulting data were used to create a similarity matrix describing the scene space in each ROI, see Figure S3 for the similarity matrices of each ROI.

A separate behavioral study asking for an explicit judgment of scene similarity was performed to examine the perceived similarity between the 100 scenes. Using this data a similarity matrix was derived that was representative of scene space as defined by perceived similarity (see Figure S2). The data was split in half to test reliability of the scores, and similarity measures across the two halves correlated with an *r* = 0.84.

**Figure 1:**
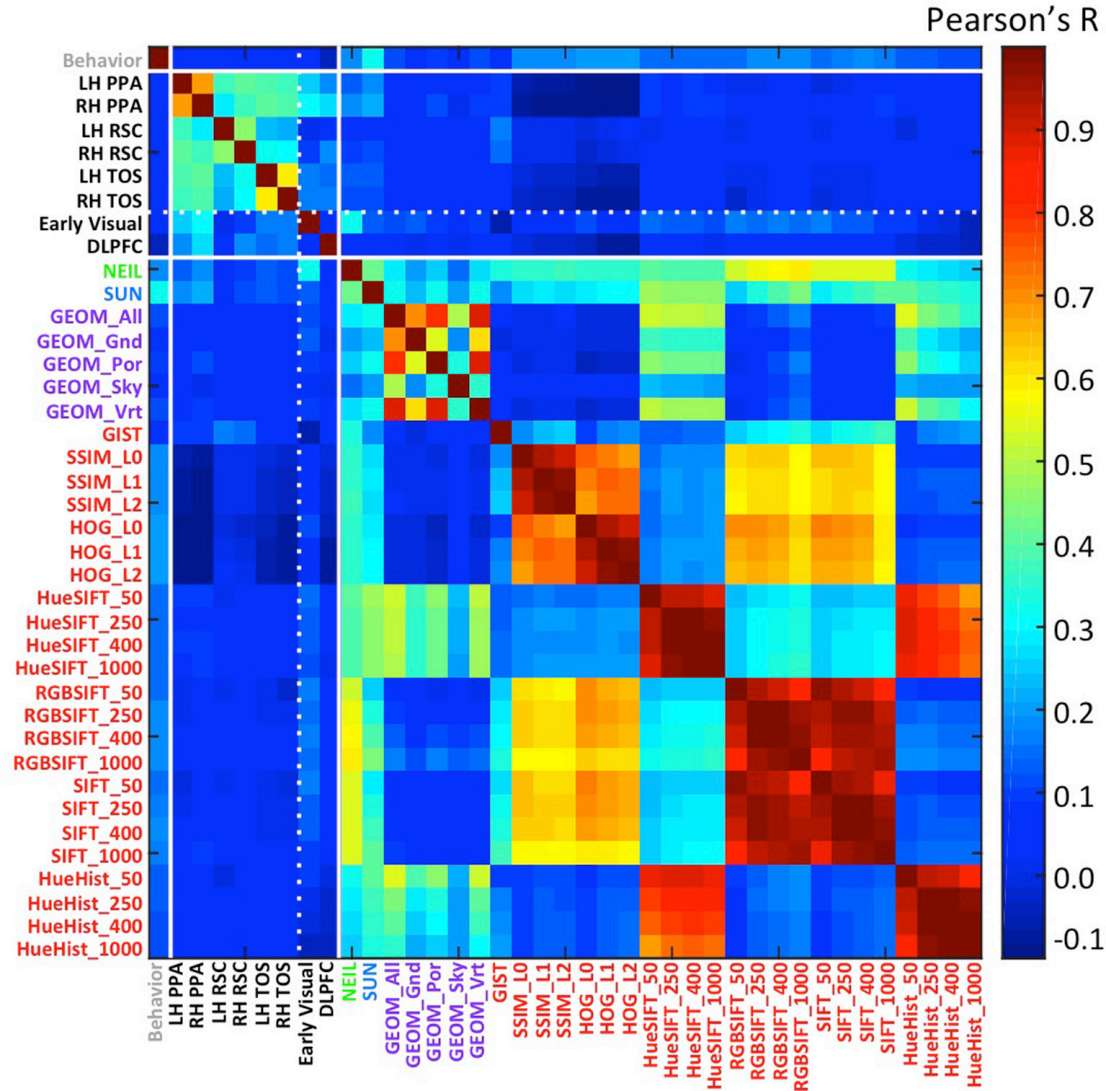
**Similarity matrix across different methods for constructing a scene space**. Each cell is the *r*-value computing the correlation between the similarity of one scene space (e.g., voxel space in LH PPA) with another (e.g., attribute space in NEIL). Scene ROIs include the PPA, RSC, and TOS for each hemisphere, and two control brain regions—an early visual region as well the DLPFC. Computer vision methods are grouped according to their nominal level of representation—e.g., GEOM is mid-level (purple); and HOG is low-level (red).

Finally, feature spaces defined through 30 different computer vision (CV) techniques were used to construct a scene space for each CV method. The features were cross-correlated for each pairwise correlation of the 100 different scenes to obtain a measure of similarity, which resulted in each similarity matrix or scene-space. Data were Fisher corrected, or log transformed (behavioral data), and correlated across the different scene-spaces to determine the similarity between these different scene representations (Figure 1; Table 1).

**Table 1:**
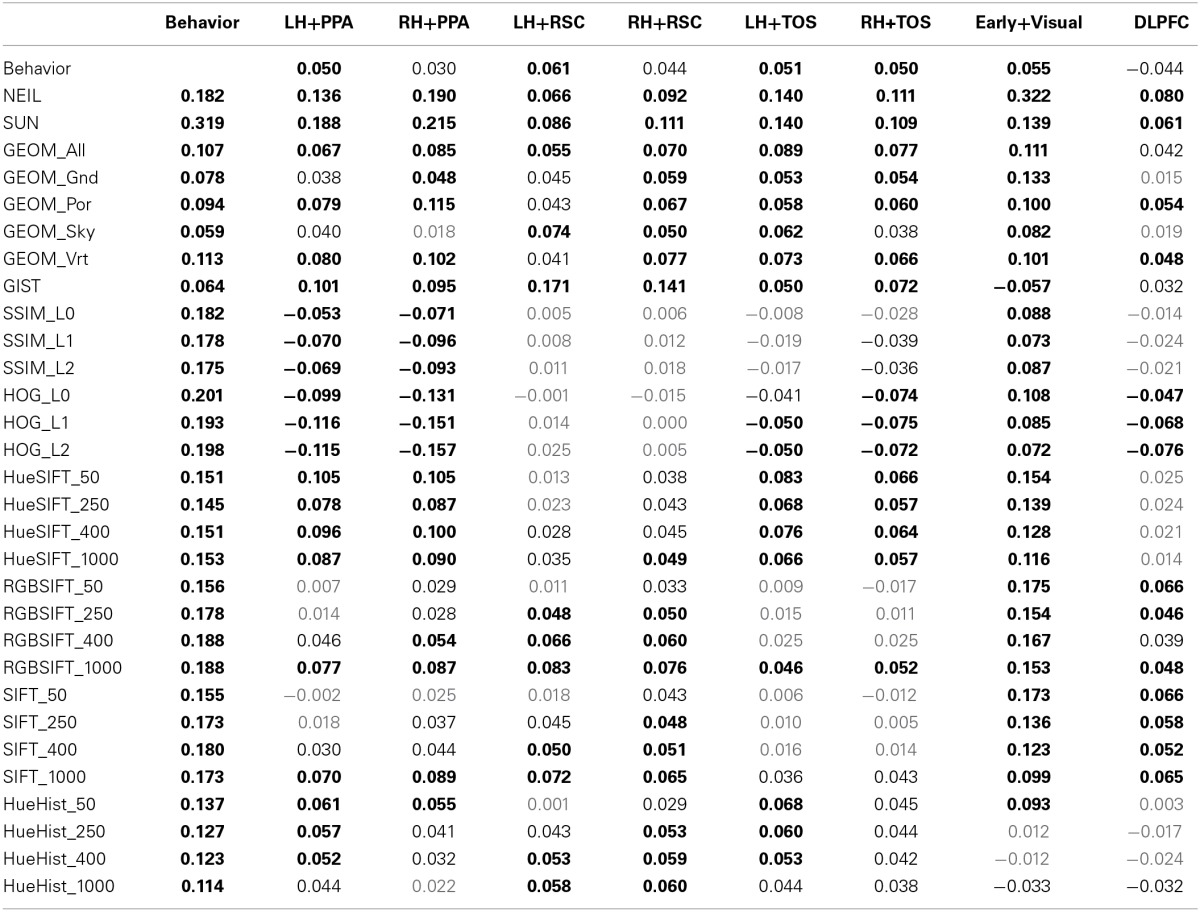
**Pearson's *r*-values for the correlations between similarity matrices**.

*Gray values indicate p >0.05; and bolded values indicate survived correction for multiple correlations*.

One of the clearest results within the similarity matrix across methods shown in Figure 1 is how much more similar the scene-selective brain regions are to themselves as contrasted with any other measure, and how similar subsets of the computer vision methods are to themselves as contrasted with either the brain or behavioral methods. One of the implications of this pattern is that we still have a ways to go in accounting for the consistent patterns of neural encoding for visual stimuli. This work-to-be-done notwithstanding, the average correlation across scene ROIs not including hemisphere correlate (e.g., LH PPA × LH RSC; LH PPA × RH TOS) was *r* = 0.34, *SD* = 0.07. The greatest similarities resulted from comparing across hemisphere of the same region (e.g., LH PPA × RH PPA); mean *r* = 0.58, *SD* = 0.11. The correlations between brain regions was considerably lower when comparing a scene ROI with a control region, mean *r* = 0.16, *SD* = 0.07, demonstrating the similarity specific to scene selective regions. CV measures were similar with themselves, *r* = 0.37, *SD* = 0.29. And the least similarity was when comparing scene brain ROIs with CV measures *r* = 0.04, *SD* = 0.06, however the correlations did get as high as *r* = 0.22, *p* < 1.5 × 10^48^ found between the RH PPA and the SUN measures. Similarity matrices derived from low-level features such as SSIM and HOG were either non-significant or negatively correlated with voxel space from scene regions, but found to be positively correlated with the early visual ROI. In general, the high-level CV methods (NEIL, SUN) significantly correlated with the scene ROIs, where, the low-level CV methods showed little correlation (although some did reach significance, see Table 1). Suffice it to say, there is a great deal of room for improvement in using CV measures to explain brain encoding of scenes. Critically, this is not due to noise in the signal—as already mentioned, there are strong correlations across the scene-selective ROIs, supporting the assumption that there is a meaningful code being used to process scenes, it just has yet to be cracked. However, that we observe significant correlations with some CV measures suggests we are making progress in explicating this code, and that the continued search for correspondences between computer vision models and patterns of brain activity may prove fruitful.

A more surprising result from our study is that correlations with brain regions was stronger with CV models (especially those with high- and mid- level features; average of SUN, NEIL, and GEOM All *r* = 0.11, *SD* = 0.05) than with behavioral similarity judgments (average *r* = 0.05, *SD* = 0.01). From these results we infer that perceived similarity between scenes is based on different visual and semantic parameters than those encoded in scene-selective ROIs. From an empirical point of view, the fact that our neurally-derived scene spaces do correlate more with some of the scene spaces derived from CV models suggests that methods drawn from computer vision offer a tool for isolating specific, and perhaps more subtle, aspects of scene representation as encoded in different regions of the human brain.

**Figure 2:**
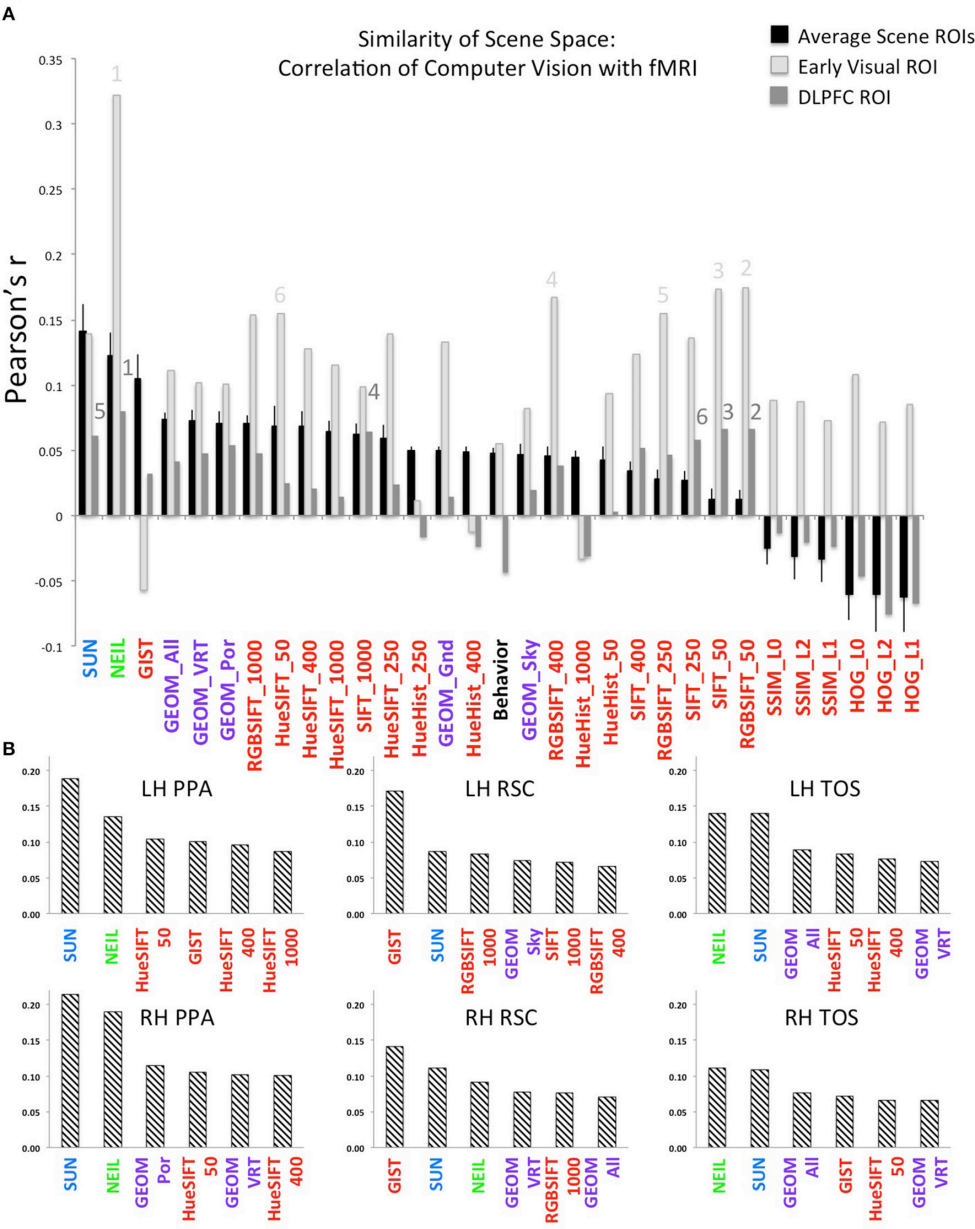
**Strength of correlation between the similarity matrix of computer vision (CV) metrics and the similarity matrix of patterns of brain activity across voxels in each ROI. (A)** The average correlation across each CV metric and each of the scene ROIs is shown by the black bars. The X-axis is ordered by the strength of this correlation. By way of comparison, the correlations between the CV metrics and the two control regions are illustrated by the light gray bars (early visual region) and the dark gray bars (DLPFC). Error bars indicate standard error across the six scene ROIs (LH and RH of the PPA, RSC, TOS). Note that font color indicates the approximate level of featural analysis implemented in each specific CV metric: blue and green are high-level; purple is mid-level; and red is low-level. Numbers indicate the top 6 correlations in the early visual regions (light gray font, above light gray bars) and in the DLPFC (dark gray front, above dark gray bars). **(B)** The top-ranked 6 CV metrics that correlated with the neurally-derived similarity matrix in each of the 6 scene-selective ROIs. The Y-axis is Pearson's *r*-value.

Beyond examining the general correspondence between CV metrics and the neural encoding of scenes, we were interested in the nature of the CV metrics offering the best correspondence and what this might reveal about the kind of information encoded in scene-selective brain regions. Interestingly, we find that CV metrics that consider high-level visual attributes, that is, SUN and NEIL, have the strongest correlation with the scene-selective ROIs (Figure 2A). In general, the lower-level CV metrics performed the worst (e.g., SSIM and HOG) and the mid-level features as defined through the GEOM faired reasonably well and were significantly correlated with scene-selective ROIs. This latter result was not unexpected in that the GEOM feature space is designed to divide a scene into those visual properties that define major features of scenes (e.g., sky). Of particular note, GEOM Por, which emphasizes material properties was significantly correlated with the responses of the PPA, a result consistent with previous studies in which it was found that the PPA is sensitive to both textures and material information (Arnott et al., 2008; Cant and Goodale, 2011). GIST was the low-level CV model that most strongly correlated with scene ROIs—primarily RSC. This result in general was not unexpected as GIST has previously been shown to be correlated with scene ROIs (Watson et al., 2014) and with scene recognition (Oliva and Torralba, 2006).

Helping validate the significance of our results, we note that the hierarchy of correlations, with decreasing correlations progressing from high- to mid- to low-level visual features was observed only in the scene-selective ROIs, but not in the two control regions (early visual and DLPFC; Figure 2A). More specifically, although NEIL produced a strong correlation in both control regions, the other high-level model, SUN, and mid-level model, GEOM, were not the most significant correlations when compared to low-level feature models (e.g., SIFT). That low-level feature models resulted in higher-ranked correlations in early visual regions as compared to high- and mid-level feature models is consistent with the central role of these brain regions in early visual processing.

To examine the consistency of this hierarchy of feature sensitivity within the six scene-selective ROIs, we examined each ROI separately and plotted the six CV metrics that showed the best correlations (Figure 2B). Both SUN and NEIL (except for the LH RSC) consistently resulted in close to the strongest correlations with our neuroimaging data. To test the significance of the model fits, a bootstrapping method was used to test for a *p* < 0.05 correcting for multiple correlations. For a full plot of all correlations, see Figure S4. Only in the LH PPA did the SUN features significantly account for more variance in brain data than NEIL features, in the other ROIs they were statistically equivalent. The PPA and the TOS both had SUN and NEIL fitting the data the best, performing significantly better than behavior, low level features such as HueHist, SIFT, RGBSIFT. In some cases the variances accounted for by HOG and SSIM, which was negatively correlated, did not significantly differ from SUN and NEIL (LH PPA, RH PPA, RH TOS). However, it is hard to interpret the significance of a negative correlation, so we provide this result with caution. Interestingly, color also seemed to be an important feature in encoding scene space. Hue SIFT, which takes into account scale invariant local features with respect to different hue maps, gave rise to scene spaces that were correlated with the neural responses measured in both TOS and demonstrated significance above a number of other models in the PPA. Although numerically midlevel features—GEOM—correlated better than low level features, significance was only reached for GEOM_por and GEOM_sky in the RH PPA, and GEOM_all in the LH TOS. On the other hand, the RSC had a different pattern of correlations. GIST showed the strongest correlation with our neuroimaging data within the RSC, fitting significantly better than all other models in the LH RSC, and all models except for the SUN features in the RH RSC. This is consistent with previous results demonstrating a correspondence for GIST with the responses of this region (Watson et al., 2014). In the LH RSC and RH RSC SUN features and RGB SIFT correlated at levels significantly over other models, and within the RH RSC NEIL also correlated significantly over and above other models. Overall, high-level feature models produced the scene spaces most consistently correlated with the scene spaces derived from scene-selective ROIs in the PPA and TOS, whereas GIST correlated the best, and the high-level SUN and NEIL features correlated next best in the RSC.

To investigate the reliability of this dataset we split the data in two (one for each session) and tested the consistency of the results. We found the correlations between the brain data with the CV measures and behavioral judgments were very consistent over the two sessions, resulting in an average *r* = 0.76, *SD* = 0.19; where the strongest consistency was in the PPA and early visual regions *r* = 0.94, *SD* = 0.02, and the lowest consistency was in the RH RSC (*r* = 0.43) and the DLPFC (*r* = 0.63). In addition, we examined the effect of including the trials that “jiggled” on the analysis, until this point all analyses include the rotated trials. We performed the analyses with and without the rotated trials, showing very little effect of including all trials in the average, the average *r*-value obtained across all ROIs with the CV measures and behavioral data across the two analyses was 0.97, *SD* = 0.02. The most notable difference in the analysis that did not include the rotated trials was an increase in the correlation with GIST. This result provides some insight into the nature of the correlation between GIST and scene ROIs, one that may be less stable than the others and therefore may not allow theoretical inference about the nature of scene representations in these brain regions.

**Figure 3:**
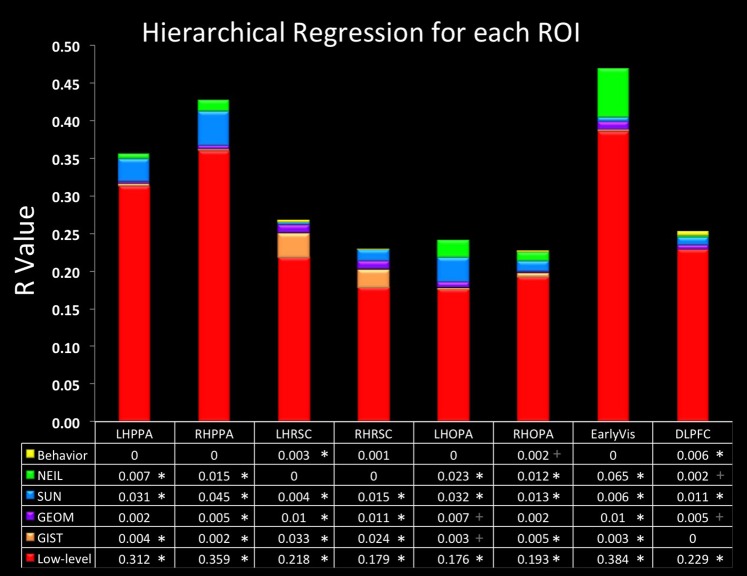
**Hierarchical regression**. Data in the bottom row of the table is the initial *R*-value yielded from the low-level CV measures. Each row above indicates the change in *R*-value when the variables listed were added. Order of blocks are (1) Low-level (HueHist, SIFT, HOG, SSIM, entered simultaneously), (2) GIST, (3) GEOM (All, Gnd, Pos, Sky, Vrt, entered simultaneously), (4) SUN, (5) NEIL, (6) Behavioral. ^*^Denotes changes in R that reached significance *p* < 0.05 corrected for multiple correlations; + denotes changes in R that reached significance *p* < 0.05 uncorrected.

Finally, we were especially interested in examining the similarity between NEIL-derived scene-space and our neuroimaging data. The web-scale nature of how NEIL learns about regularities across scene categories is appealing in that it seems to best capture both the evolutionary history of our visual systems and the kind of neural statistical learning that seems to emerge over a lifetime of experience. NEIL's features capture the visual regularities that give rise to semantic information, helping to define the visual features that give rise to scene understanding. Table 1 shows that the scene space derived from NEIL's attributes is significantly correlated with our neurally-derived scene space within each scene-selective ROI. However, the question remains about how well does NEIL do over and above all the other CV measures. To address this, we ran a hierarchical regression for each ROI (Figure 3). In this regression the first input was the low-level CV metrics (Hue Histogram, SIFT, HOG, SSIM) and the second input was to separately add GIST, to see what variance was left over when the low-level visual features were removed. Next we entered the GEOM metrics, followed by the SUN attributes, followed by NEIL, and, finally, the last block being our behavioral data. This regression demonstrates that NEIL accounts for a significant amount of the variance in defining the neurally-derived scene space over and above any of the other CV metrics in both the PPA and the TOS, as well as in early visual regions. As such, it appears as if NEIL is capturing something unique about scene representation within the PPA, TOS, and early visual regions that is not captured by any of the other models. The behavioral data only accounted for unique variance above that already accounted for in the LH RSC and the DLPFC.

## Discussion

We started with the challenge of specifying the “language” of mid- and high-level features supporting object and scene recognition. Given the large space of possible answers to this question, we attempted to constrain the possible answers by applying a variety of computer vision models that make somewhat different assumptions regarding the nature of this language. To evaluate the effectiveness of these different assumptions, we explored the degree to which each model accounted for patterns of neural data arising from scene processing by scene-selective brain regions. We found that:

- The NEIL and SUN models—both of which rely on mid- and high-level visual features—were best at accounting for variation in the neural responses of both the PPA and the TOS. The fact that NEIL was equivalent to SUN indicates that statistically-derived features offer a viable model of scene representation that may, ultimately, reveal non-intuitive coding principles for scenes.
- The GIST model—a model which relies on global spatial properties of scenes—was best at accounting for variations in the neural responses of the RSC. Additional unique variance in the RSC was accounted for by our behaviorally-obtained similarity ratings.
- Given points (1) and (2), there is support for a model of scene processing in which PPA and TOS are coding scene information differently from RSC, with the former coding for the visual attributes within scenes and the latter coding for higher-order, scene categories.
- The most effective computer vision models were better than behaviorally-obtained ratings of scene similarity at accounting for variance in our neural data.

Of note, we found that regions of the brain selective for scene processing respond similarly to the same scenes, and treating, similar scenes as defined in one ROI as similar in another ROI, and, different scenes as defined in one ROI as different in another ROI. This pattern of results suggests that there is a stable encoding pattern for scenes within scene-selective brain regions and that voxel-to-voxel variation carries meaningful information regarding commonalities and differences between scenes.

These results suggest that, as a first step, applying computer vision models to neural data may allow us to better understand how scene information is encoded in neural systems. In particular, we view the application of NEIL as having the most promise in that its “vocabulary” of scene attributes does not ultimately depend on intuition, but rather on those regularities that can be learned from scene data. By way of example, NEIL includes visual features such as textures, color/shape combinations, and geometric configurations that do not readily correspond to any typical part label, but that may help enable NEIL's ability to categorize scenes. More generally, models such as NEIL offer better-specified theories of visual representation: it is our contention that NEIL and other artificial vision models offer meaningful—and testable—constraints at multiple levels of visual processing. With respect to our present results, we can now iterate toward more fine-grained tests of the most promising models (NEIL, SUN, GIST).

Beyond the well-specified representational constraints inherent in any functional model of computer vision, adopting multiple models also allowed us to consider a range of feature representations. In particular, the computer vision methods employed here ranged from analyzing low-level features, such as orientation information and spatial frequency, to high-level features, such as semantic categories. As expected, the low-level feature spaces (e.g., SIFT) were best correlated with patterns of voxel activity found in early visual brain regions, but were not highly positively correlated with the patterns of activity arising from scene-selective cortex. In contrast, as discussed, NEIL, SUN, and GIST gave rise to feature spaces that were most strongly correlated with the patterns of activity arising from scene-selective brain regions. Moreover, we found that NEIL's feature space, in particular, accounted for unique variance that could not be accounted for by any of the other methods. Together, our results indicate that the PPA, RSC, and TOS are involved in the processing of mid- to high-level features of scenes. We should note also that one curious result is the fact that NEIL accounted for significant variance in early visual areas. However, without a map of retinotopy for these early visual areas, it is difficult to say much about what NEIL's features may reveal about these processing areas.

Finally, we also observed that two models relying primarily on low-level features were significantly correlated with certain scene-selective brain regions. First, GIST correlated quite strongly with the RSC, replicating previous findings demonstrating a connection between GIST and the RSC functional properties (Watson et al., 2014). This suggests that the RSC may contribute to processing an image's spatial envelope or global scene properties which are known to be involved in scene understanding (Oliva and Torralba, 2006; Greene and Oliva, 2009). Moreover the RSC has been shown to process a representation of the scene that is abstracted from what is seen in the environment, typically processing a broader environment that extends beyond the current saccade (Epstein and Higgins, 2007; Park et al., 2007; Park and Chun, 2009). One possibility is that the RSC may process the low spatial frequencies or global properties of a scene that are strongly indicative of scene category. In addition, RSC was found to correlate with behavioral ratings of similarity, which was not found in the PPA or the TOS. That the correlations with GIST and behavior were unique to the RSC may suggest that RSC may provide a more categorical, or high-order representation of scenes. The second low-level model proved to be important were SIFT features in color domains that correlated strongly with multiple scene-selective regions: Hue SIFT showed strong correlations with the PPA and TOS, while RGB SIFT showed strong correlations with the RSC. In earlier work, junctures within scenes, which may be similar to SIFT features, were found to be important for scene categorization (Walther and Shen, 2014). Our results add to this finding by suggesting that key features but specifically within different color domains also carry information regarding scene categories. That is, scene-selective brain regions may rely on color cues in scene understanding—a claim consistent with earlier behavioral research on scene processing (Oliva and Schyns, 2000). At the same time, the lower correlations observed for the Hue Histogram model as compared to the Hue SIFT and RGB SIFT models suggest that it is not color *per se* that carries this information, but rather information about scene categories arises from an interaction of SIFT features within color domains. In particular, the perirhinal cortex—a region of the parahippocampal gyrus adjacent to the PPA—has been shown to unitize properties across an object; for example, that stop signs are red (Staresina and Davachi, 2010). As such, this function may extend to the parahippocampal region more generally being seen as unitizing diagnostic features, with the PPA supporting this function within scene processing.

In sum, we explored the visual dimensions underlying the neural representation of scenes using an approach in which models derived from computer vision are used as proxies for any psychological theory. While this approach may seem somewhat indirect, we argue that it is a necessary precursor in that extant psychological models have typically been somewhat underspecified with respect to the potential space of visual features. Humans can identify scenes effortlessly under a wide variety of conditions. For example, we can name scenes with near-equivalent accuracy when shown both photographs and line drawings, and with color present or absent. There is, then, no single feature dimension that drives the organization of scene-selective cortex. However, some dimensions are likely to prove more effective than others. Color is just one example of the many diagnostic cues that are used to aid in scene perception. There are almost surely a range of visual attributes and their associations within scenes that are diagnostic as to their categories and to which we are sensitive (Bar et al., 2008; Aminoff et al., 2013). Computer vision models, to the extent that they make representational assumptions with respect to scene attributes and their associations (i.e., models with a less well-understood representational basis may not actually be particularly informative), are, therefore, useful for better explicating those featural dimensions involved in human visual scene processing.

### Conflict of interest statement

The authors declare that the research was conducted in the absence of any commercial or financial relationships that could be construed as a potential conflict of interest.
