# Supplementary material for: Applying artificial vision models to human scene understanding
Source: Front Comput Neurosci. 2015 Feb 4;9:8. doi: 10.3389/fncom.2015.00008 (PMC4316773; doi:10.3389/fncom.2015.00008)

Supplementary Material
Applying artificial vision models to human scene understanding

Elissa M. Aminoff, Mariya Toneva, Abhinav Shrivastava, Xinlei Chen, Ishan Misra, Abhinav Gupta, & Michael J. Tarr

Front. Comput. Neurosci., doi: 10.3389/fncom.2015.00008

Methods, Stimuli:
Scene Categories:

Aquarium
Arrival Gate Outdoor
Auto Factory
Basketball Arena
Bleachers Indoor
Bay Window Outdoor
Canyon
Car Dealership
Cargo Container Interior
Cargo Deck Airplane
Coast
Conference Room
Cornfield
Desert Road
Desert Vegetation
Editing Room
Elevator Indoor
Garbage Dump
Gulch
Hallway
Industrial Area
Manhole
Music Store
Nightclub
Nunnery
Ocean
Oilrig
Oyster Bar
Parade Ground
Parking Garage Indoor
Parking Garage Outdoor
Picnic Area
Planetarium Indoor
Podium Outdoor
Rainforest
Security Checkpoint
Sewer
Snowfield
Stage Indoor
Stone Circle
Strip Mall
Subway Station Corridor
Swamp
Swimming Hole
Theater Outdoor
Track Outdoor
Underwater Wreck
Volleyball Court Outdoor
Waterfall Cascade
Wine Cellar Bottle Storage

Scene Stimuli:

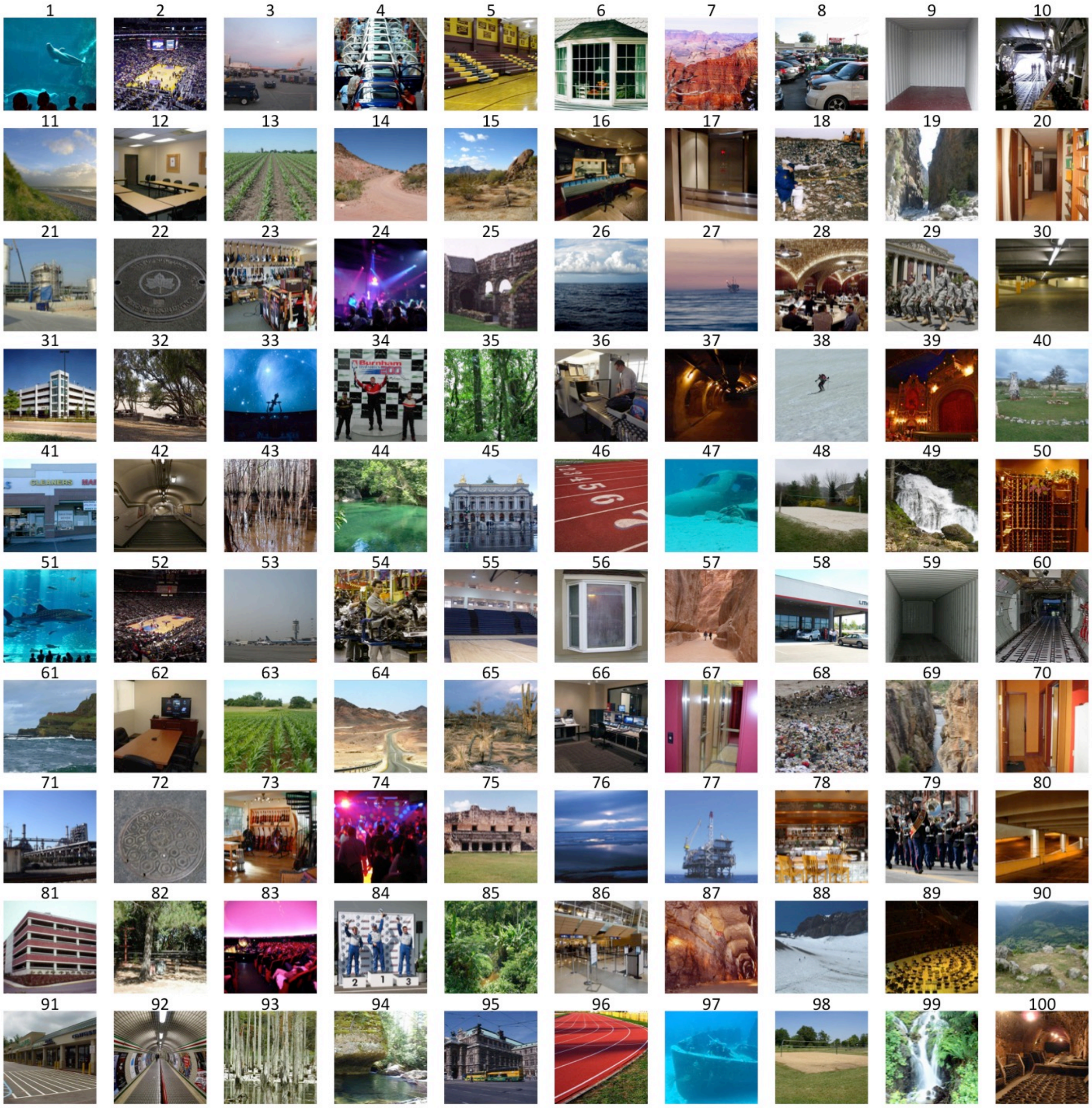

Figure S1: 100 scene stimuli used in the experiment. Numbers refer to the stimuli ID references in the similarity matrices presented in Figures S2-S3.

Results:

Figure S2: Behavioral judgments of perceived similarity from a scale of 1 (completely different) to 8 (identical). Each row/column represents a scene, numbers refer to stimuli ID as listed in Figure S1. Each cell represents the average response of each pairwise comparison.

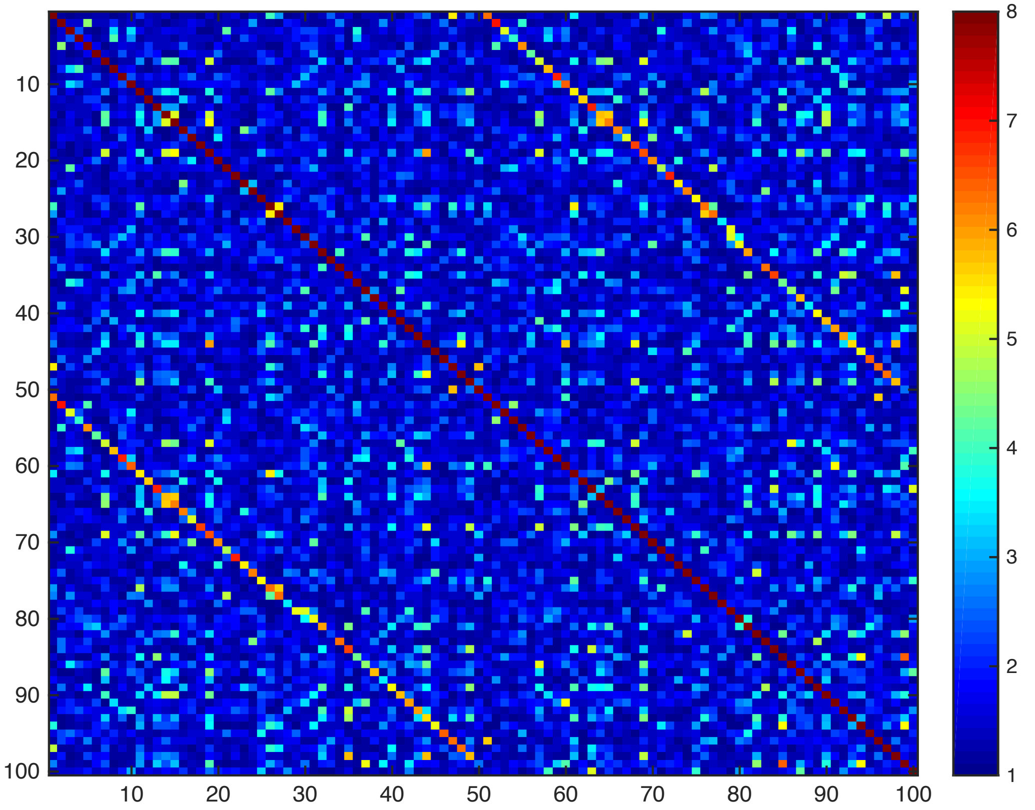

Figure S3A-H: Similarity matrices for each ROI, each row/column represents a scene, numbers refer to stimuli ID as listed in Figure S1. Each similarity matrix is the average of similarity matrices across participants. Cell values are Pearson's *r*. Stimuli were not ordered in any meaningful grouping in that we did not make any *a priori* assumptions regarding the organization of scene-space.

A) LH PPA

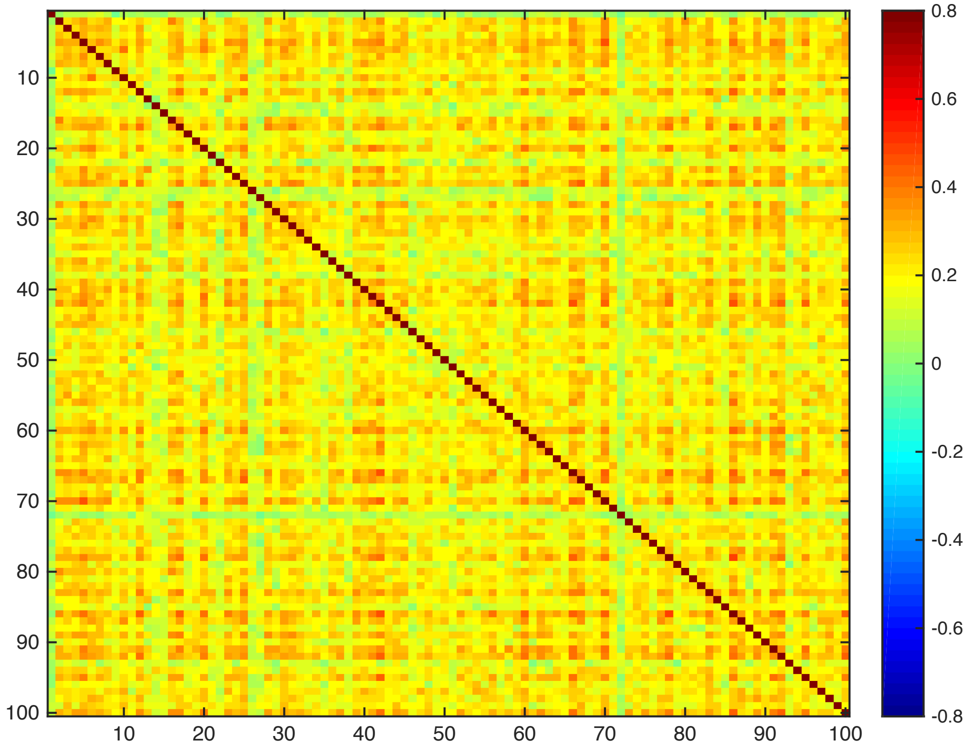

B) RH PPA

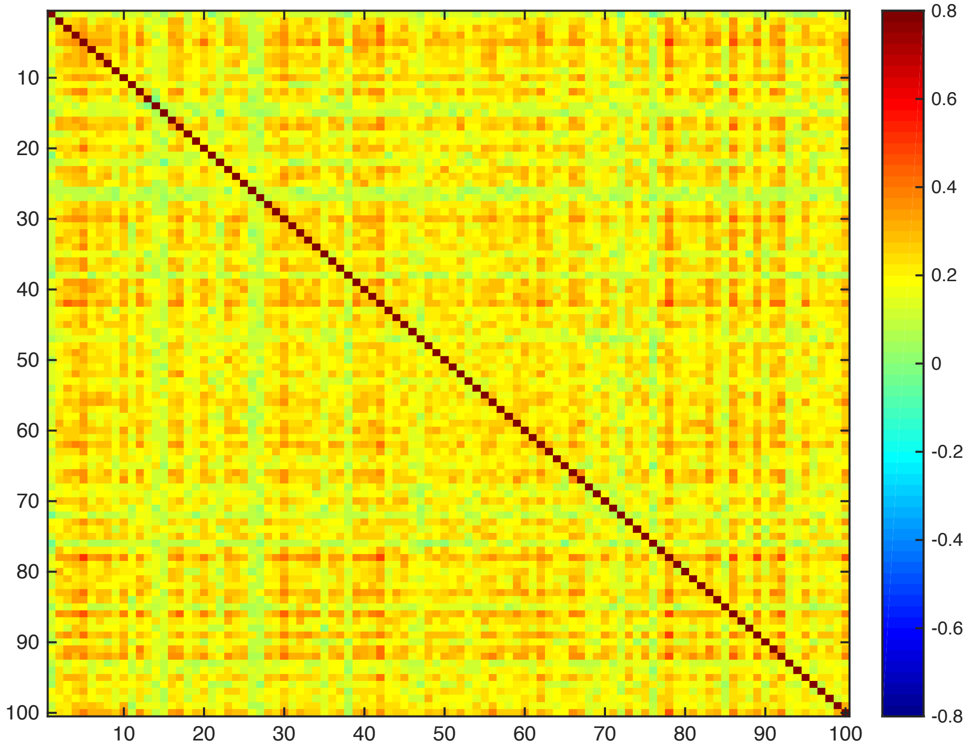

C) LH RSC

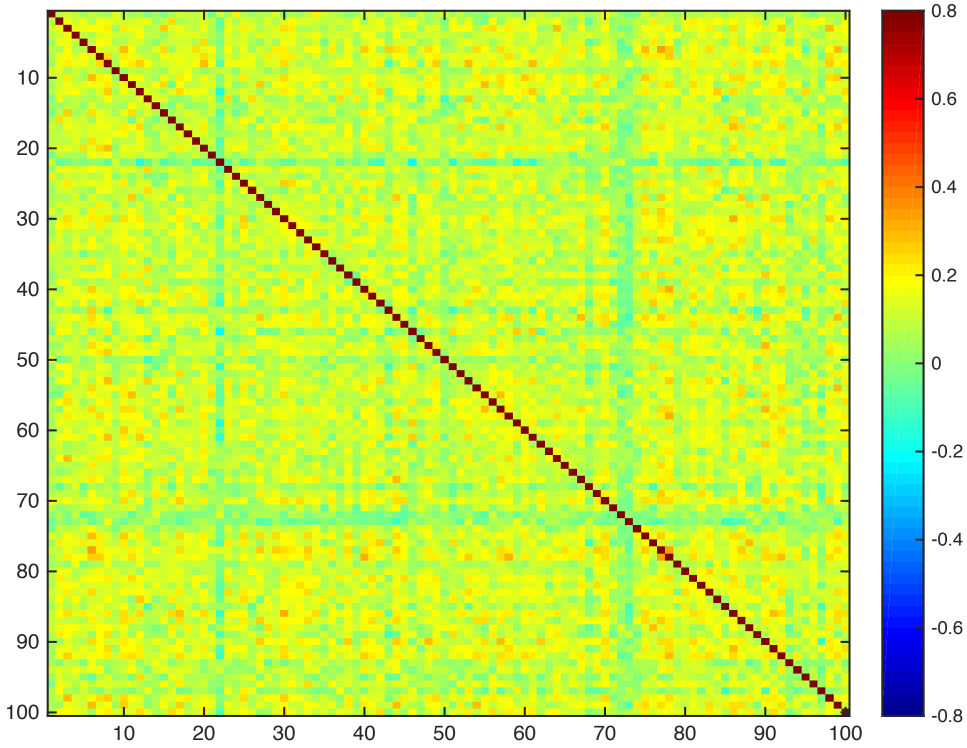

D) RH RSC

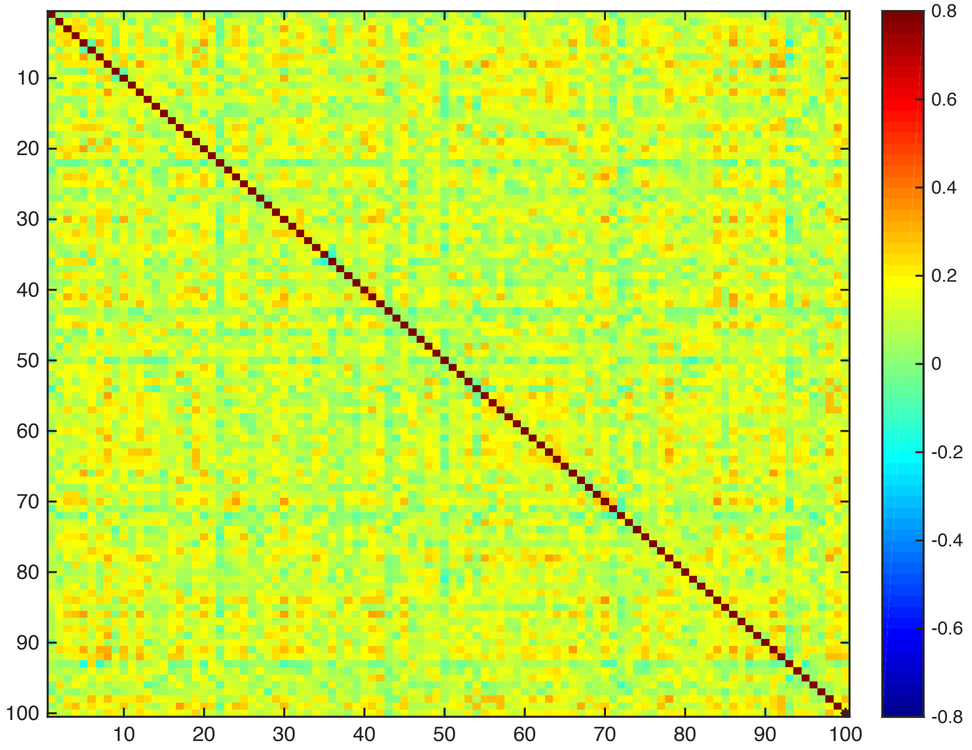

E) LH TOS

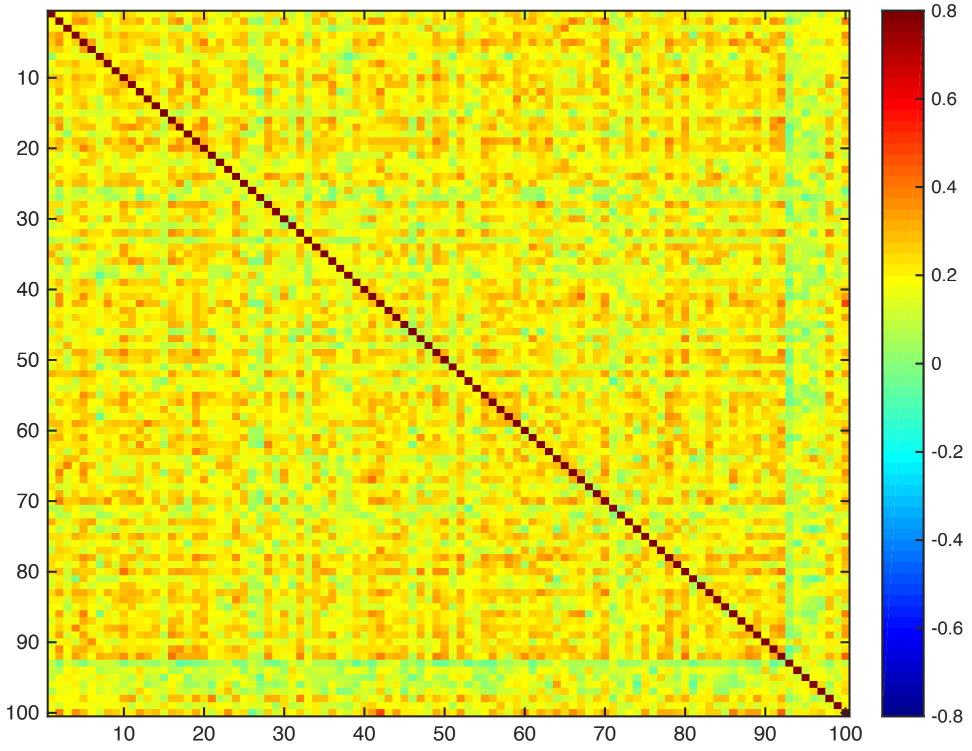

F) RH TOS

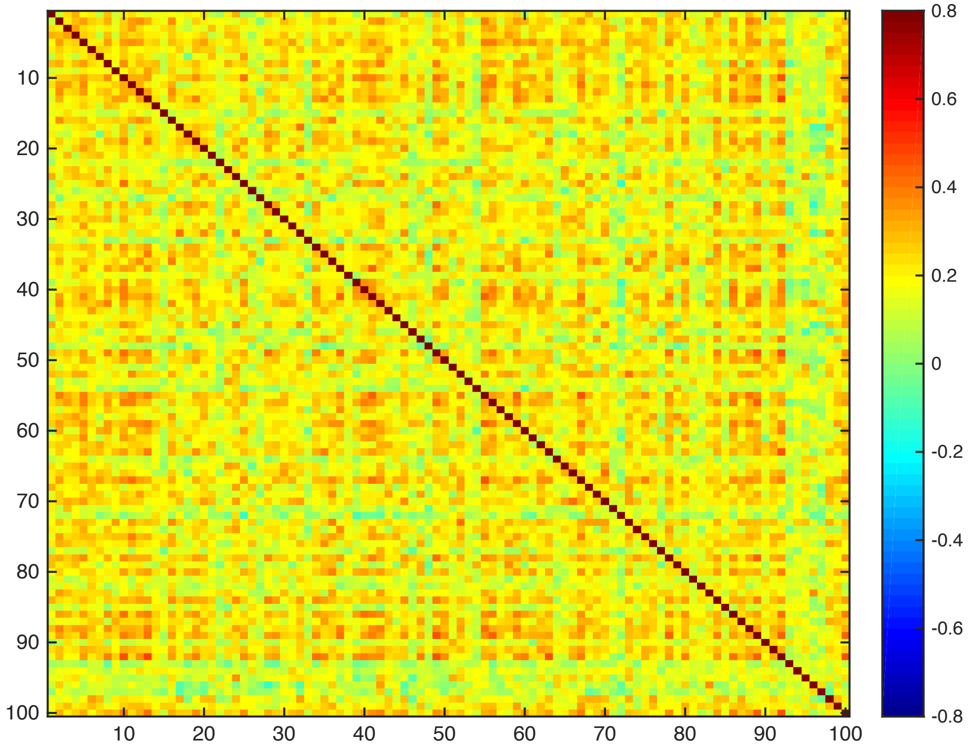

G) Early Visual

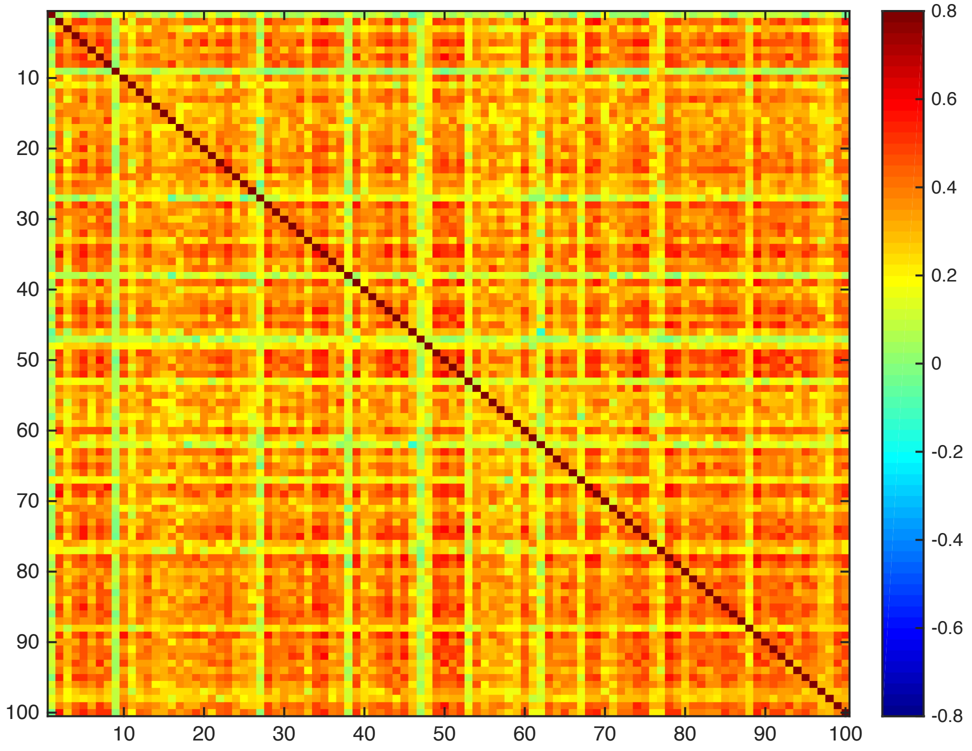

H) DLPFC

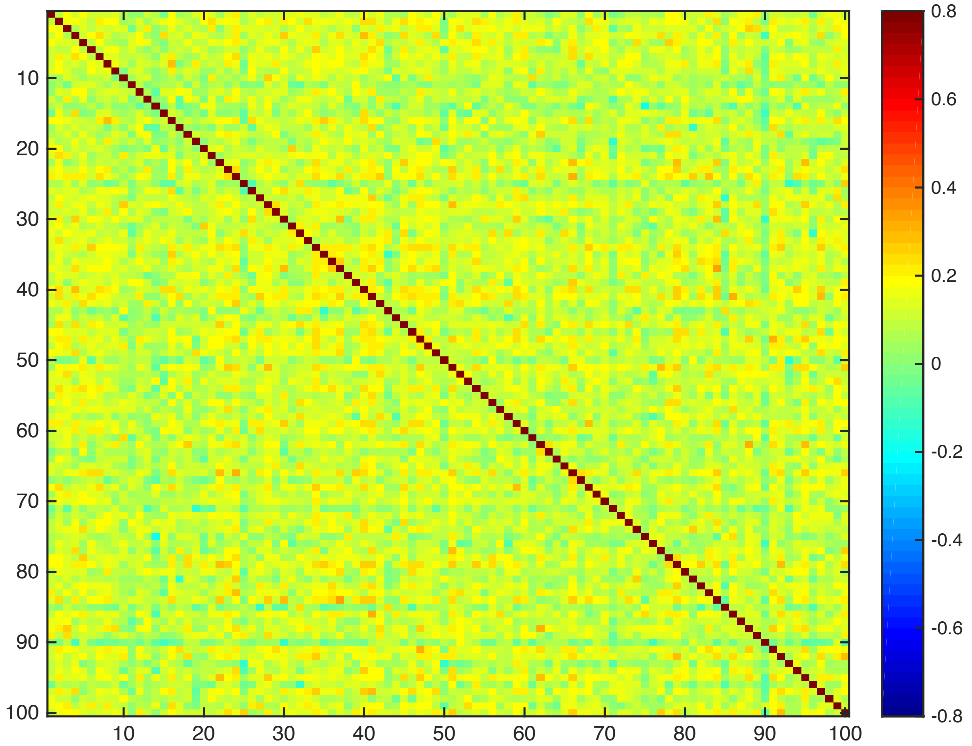

Figure S4: Testing statistical significance of model fits using a bootstrapping method. Model 1 – rows – was tested for better fit relative to Model 2 – columns. Green indicates that Model 1 was significantly better than Model 2, $p < .05$. Pink indicates Model 1 was significantly better than Model 2, $p < .05$, but in a negative direction with the neural data.

LH PPA

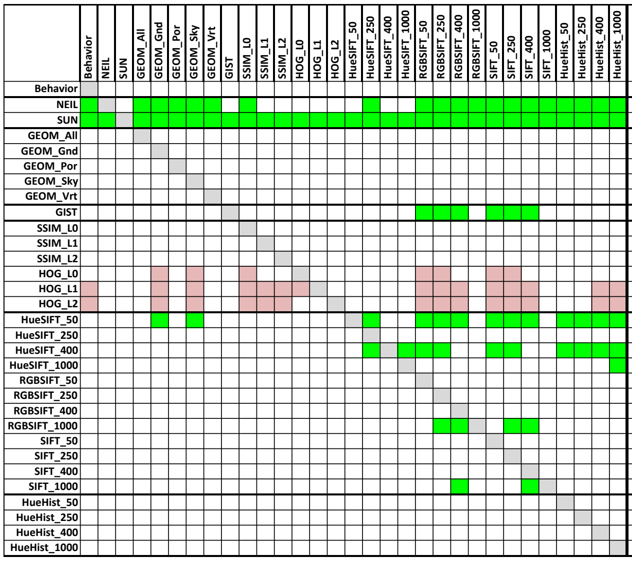

RH PPA

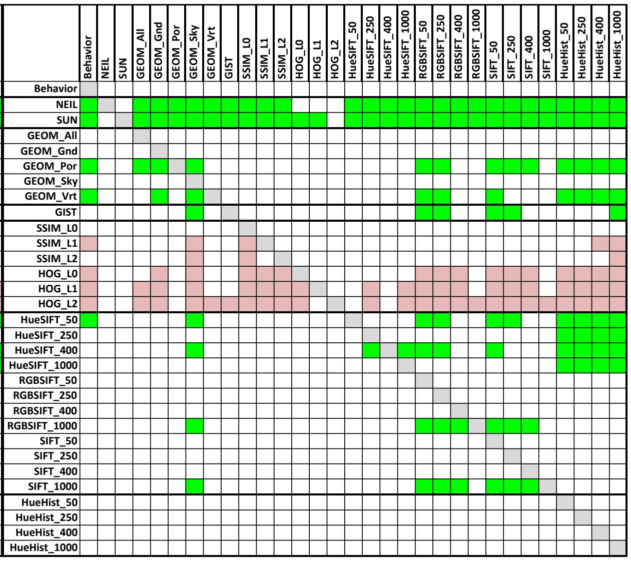

LH RSC

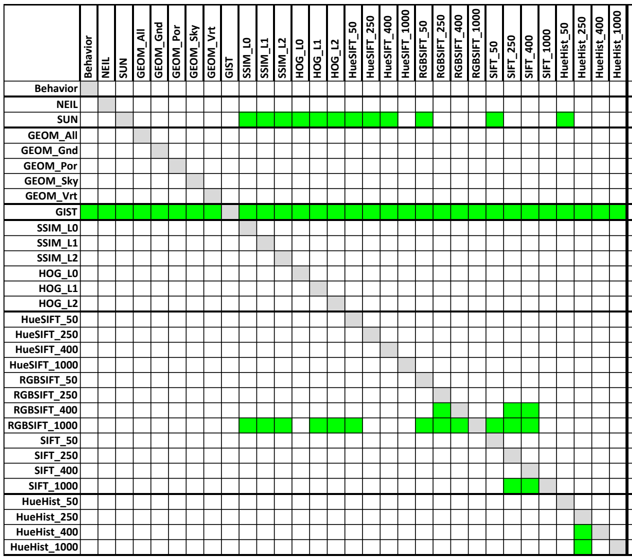

RH RSC

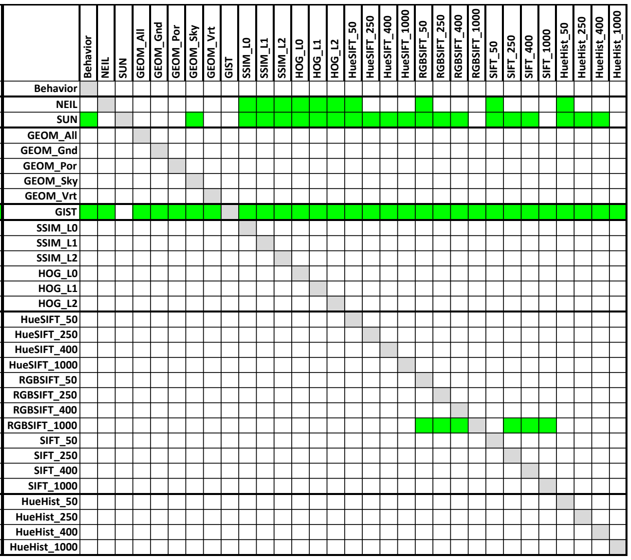

LH TOS

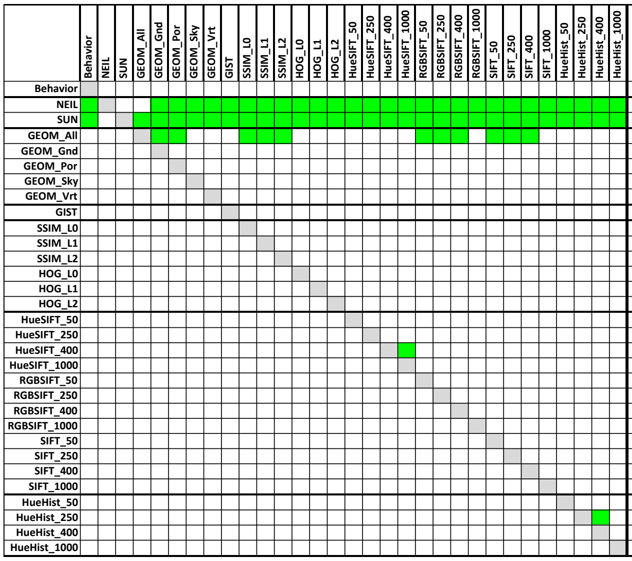

RH TOS

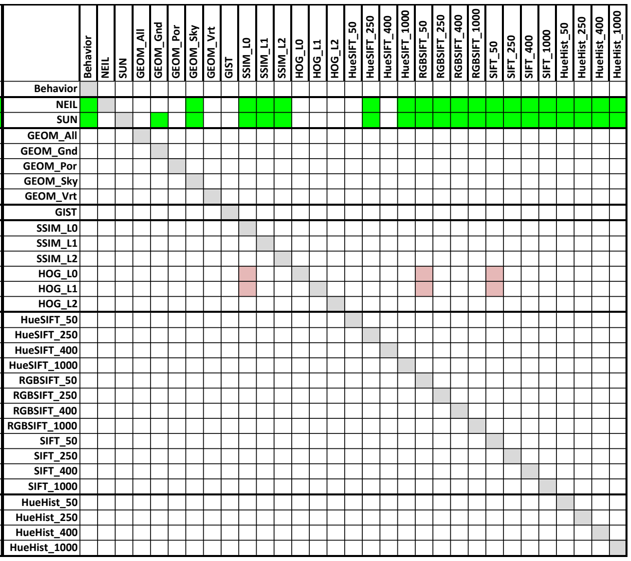

Early Visual

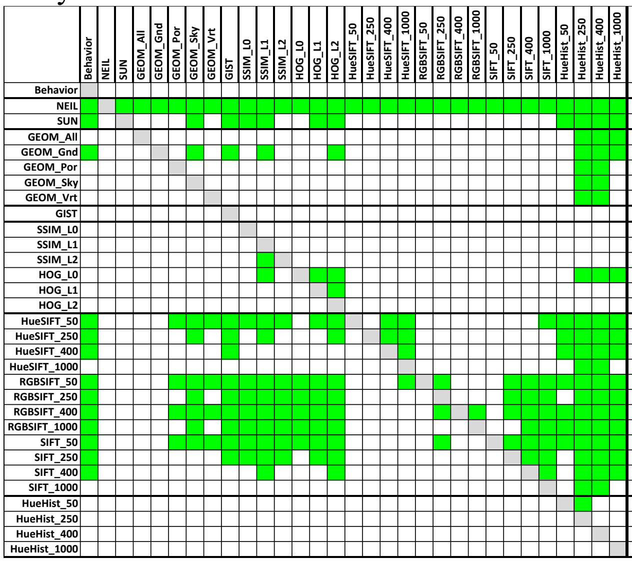

DLPFC

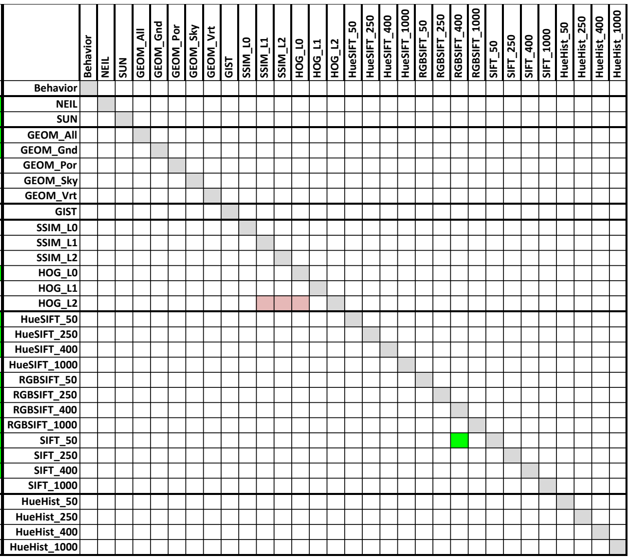

Supplement: Supplementary file 1 [file DataSheet1.PDF]
